# NetGenes: A Database of Essential Genes Predicted Using Features From Interaction Networks

**DOI:** 10.3389/fgene.2021.722198

**Published:** 2021-09-23

**Authors:** Vimaladhasan Senthamizhan, Balaraman Ravindran, Karthik Raman

**Affiliations:** ^1^Centre for Integrative Biology and Systems mEdicine (IBSE), Indian Institute of Technology (IIT) Madras, Chennai, India; ^2^Robert Bosch Center for Data Science and Artificial Intelligence (RBCDSAI), IIT Madras, Chennai, India; ^3^Department of Computer Science and Engineering, IIT Madras, Chennai, India; ^4^Department of Biotechnology, Bhupat and Jyoti Mehta School of Biosciences, IIT Madras, Chennai, India

**Keywords:** essential genes, networks, machine learning, interaction network, database

## Abstract

Essential gene prediction models built so far are heavily reliant on sequence-based features, and the scope of network-based features has been narrow. Previous work from our group demonstrated the importance of using network-based features for predicting essential genes with high accuracy. Here, we apply our approach for the prediction of essential genes to organisms from the STRING database and host the results in a standalone website. Our database, NetGenes, contains essential gene predictions for 2,700+ bacteria predicted using features derived from STRING protein–protein functional association networks. Housing a total of over 2.1 million genes, NetGenes offers various features like essentiality scores, annotations, and feature vectors for each gene. NetGenes database is available from https://rbc-dsai-iitm.github.io/NetGenes/.

## 1. Introduction

Essential genes are indispensable to organisms for their growth and reproduction. The deletion of these genes will either compromise an organism's viability or result in a profound loss of fitness (Rancati et al., 2018). Classification of genes as essential and non-essential is challenging since the essentiality of a gene depends on a variety of factors (Zhang et al., 2016). Various computational approaches have been devised to predict essential genes, and most of them use sequence-based features for training the model (Song et al., 2014; Liu et al., 2017; Nigatu et al., 2017). A few studies have included network-based features in their machine learning (ML) model, but only alongside sequence-based features (Hwang et al., 2009).

Our previous work (Azhagesan et al., 2018), hereafter referred to as “original paper”, utilized a purely network-based feature set to predict gene essentiality. Essential genes for 27 bacterial organisms were predicted using features extracted from protein–protein interaction networks. The 27 interactomes used in the original paper were phylogenetically diverse; this ensures that the cohort is representative of a large class of bacterial interactomes. The model showed considerable predictive power even when it was tested on genes from an unseen organism.

Here, we extend our previous research by using the same 27 phylogenetically diverse interactomes to predict gene essentiality for a much larger array of bacterial networks. Retrieving 2,711 bacterial interactomes from STRING 11 (Szklarczyk et al., 2019), a graph mining method called Recursive Feature Extraction (ReFeX) (Henderson et al., 2011) was employed in engineering the features from the interactomes. Using the dataset from the original paper as the training set, we predicted the essential genes for each of the bacterial interactomes. Our results are available via NetGenes, a standalone web database.

## 2. Methods

### 2.1. Interactome Data Collection and Feature Engineering

STRING (https://string-db.org/) hosts one of the largest collections of protein–protein interactomes (interaction networks). An interactome draws edges between pairs of functionally associated proteins and it includes almost all proteins in an organism to form a single huge network. These interactomes provide information about known and predicted interactions and functional associations among proteins in a given organism. A total of 5,090 interactomes available in STRING version 11.0 were first retrieved. The Environment for Tree Exploration (ETE) Toolkit is a Python framework built for the analysis and visualization of phylogenetic trees (Huerta-Cepas et al., 2016). NCBI taxonomy analysis offered by the ETE library was used to classify the STRING interactomes by phyla. Interactomes belonging to different phyla in Kingdom Bacteria were separated from the cohort and used for our essential gene predictions. From the 5,090 interactomes, a final dataset comprising 2,711 bacterial interactomes was used for building the model.

The main intent of original paper was to ascertain if network-based features, such as centrality measures, can outperform sequence-based features, such as length of sequence, amino acid composition and GC content, in predicting gene essentiality. For comparison, the model from the original paper was compared with a previous study that used sequence-based features to identify essential genes (Liu et al., 2017). The results from the original paper (Azhagesan et al., 2018) proved that the models using purely network-based features can perform better than sequence-based features. Moreover, it was also shown that combining sequence-based and network-based features can further marginally improve the quality of predictions.

**Figure 1:**
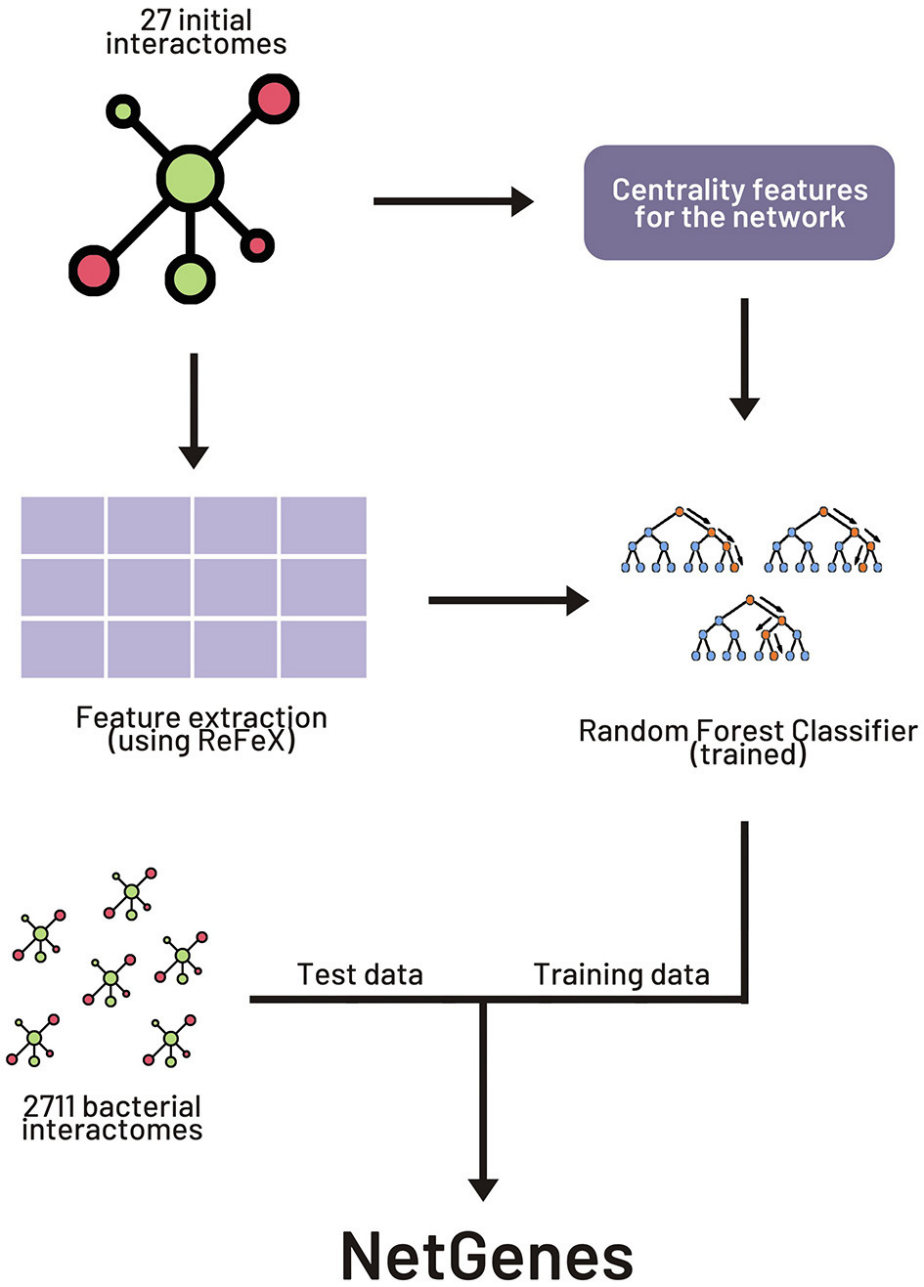
Workflow for creating NetGenes database. The initial 27 interactomes were used as the training dataset to build the machine learning (ML) model. The 2711 interactomes were run through the ML model to obtain the essential gene predictions. These predictions are curated and published in the “NetGenes” database.

The original paper experimented with various combinations of network-based and sequence-based models. We here focus on the widely applicable purely network-oriented features, and therefore we used the “283 network” variant of the feature set stated in the original paper. This set includes a number of features including “ReFeX” features. ReFeX is a feature extraction algorithm that recursively combines local and neighborhood features of a given network and outputs “regional” features that capture network behavior (Henderson et al., 2011). This feature extraction algorithm was applied on all the interactomes. In order to replicate the performance of the original paper, the 267 ReFeX features employed in the article were retrieved from the extracted features. Along with these 267 features, 12 centrality measures, clique number, clustering coefficient, biconnected components, and weighted degree were added to the feature matrix, resulting in the total of 283 features. A list of these features can be found in the Supporting Information section of the original paper. Figure 1 illustrates the basic workflow for the building of NetGenes.

### 2.2. Building the ML Model

For the training dataset, interactomes of the 27 species (see Table 1) used in the original paper were taken, and their features were computed to form the feature matrix. All 27 interactomes contained at least 50,000 edges and hence the features extracted from them will be sufficient for a generalizable model. Supplementary Table 1 outlines the statistics of these 27 interactomes. The labels for essential and non-essential genes were taken from the Database of Essential Genes (DEG) (Luo et al., 2014). All the genes present in DEG are considered as essential genes in our dataset, and all other genes are taken as non-essential genes. After mapping the DEG labels to the protein IDs in the 27 interactomes, the final training data consisted of 8,754 essential genes and 74,492 non-essential genes. Random Forest Classifier implementation from the sci-kit learn package (Pedregosa et al., 2018) was used as the ML algorithm. In order to find the optimal adaptation parameters for Random Forest, we performed 10-fold hyper-parameter optimization using Grid Search method available in sci-kit learn package. The optimal parameter set was found to be {number of trees: 150, criterion: “entropy”, max. features: “sqrt”}.

**Table 1:** Table showing LOSO AUROC scores for DEG10 and DEG15 datasets.

**Organisms**	**AUROC-DEG10**	**AUROC-DEG15**
*Acinetobacter sp*. ADP1	0.83	0.87
*Burkholderia pseudomallei* K96243	0.65	0.72
*Bacillus subtilis*	0.87	0.90
*Burkholderia thailandensis* E264	0.90	0.95
*Bacteroides thetaiotaomicron* VPI-5482	0.72	0.70
*Escherichia coli* K-12 substr MG1655	0.85	0.90
*Caulobacter crescentus* NA1000	0.91	0.91
*Campylobacter jejuni*	0.66	0.67
*Francisella tularensis novicida* U112	0.75	0.81
*Haemophilus influenzae*	0.53	0.65
*Helicobacter pylori* 26695	0.59	0.67
*Mycoplasma genitalium*	0.62	0.82
*Mycoplasma pulmonis*	0.81	0.75
*Pseudomonas aeruginosa* UCBPP-PA14	0.77	0.76
*Pseudomonas aeruginosa*	0.67	0.81
*Porphyromonas gingivalis* ATCC 33277	0.77	0.88
*Mycobacterium tuberculosis* H37Rv	0.74	0.90
*Staphylococcus aureus* NCTC 8325	0.82	0.92
*Staphylococcus aureus* N315	0.85	0.70
*Shewanella oneidensis*	0.88	0.80
*Streptococcus pneumoniae* R6	0.72	0.92
*Streptococcus pyogenes* NZ131	0.85	0.74
*Streptococcus sanguinis* SK36	0.92	0.86
*Salmonella typhimurium* LT2	0.70	0.95
*Sphingomonas wittichii* RW1	0.82	0.72
*Salmonella enterica* serovar Typhi Ty2	0.89	0.72
*Vibrio cholerae*	0.63	0.85
*Acinetobacter baumannii ATCC 17978	0.69	0.79
*Burkholderia cenocepacia J2315	0.72	0.79
*Campylobacter jejuni 81176	0.78	0.82
*Mycobacterium tuberculosis H37Rv II	0.72	0.80
*Mycoplasma pneumoniae M129	0.81	0.72
*Ralstonia solanacearum GMI1000	0.79	0.78
*Rhodopseudomonas palustris CGA009	0.75	0.69

Organisms prefixed with “*” are exclusive to the DEG15 dataset.

The validation method adopted was “leave-one-species-out” (LOSO), where we trained the model on all species but one, and tested its performance on the remaining (one left out) species. There existed a huge imbalance between the positive labels (essential genes) and negative labels (non-essential genes); therefore, the dataset sampler from “pandas” library was used to under-sample the negative dataset (McKinney, 2010; The Pandas Development Team, 2020). A 10-fold cross-validation was employed to increase the robustness of the model and ensure that all the negative labels were featured at least once in the training dataset. AUROC score is used as the scoring metric for the model and it is calculated using sci-kit learn package. Statistical tests were performed using scipy package (Virtanen et al., 2020).

## 3. Results

### 3.1. Predictions on DEG15 Dataset Illustrate Model Generalizability

Recently, DEG released an update, DEG 15 (Luo et al., 2021), with an increase in essential gene labels and also including seven newer organisms. To assess the generalizability of our model, we performed two experiments. In the first experiment, we used the DEG10 labels to build a classifier, while in the second, we used the updated DEG15 labels to build a classifier. In both cases, we made predictions on all 34 organisms, as indicated in Table 1. The changes in DEG15 increased our model's AUROC by 4% on average per organism; yet, a *t*-test between the AUROC scores for DEG10 and DEG15 datasets showed that the difference was not statistically significant (*p* = 0.356). In practice, increasing the dataset size boosts the variance and, in turn, reduces the classification capability of the model (L'Heureux et al., 2017). But the fact that the change in model performance is not statistically significant, even when the dataset size increased by ≈29,200 data points (≈90,400 genes in DEG10 vs. ≈119,600 genes in DEG15, as illustrated in Supplementary Table 1) shows that our model has excellent generalization capacity. Overall, the average LOSO AUROC for the 27 organisms in DEG10 dataset was 0.77. We retained this model for predictions in the NetGenes database, since the increase in AUROC was not substantial for DEG15.

### 3.2. The NetGenes Database

The results obtained from the model are cleaned, compiled organism-wise, and converted to a comma-separated values (CSV) format. These files are hosted as a web-database called “NetGenes”. The HTML files are created in-house and hosted as GitHub Pages (https://pages.github.com/).

The complete database contains predictions for 2,163,702 essential genes spread across 2,711 bacterial organisms. The homepage is equipped with pagination and hosts a dynamic search bar and download links for each organism. An “Individual species” page contains a table of all predicted essential genes for the particular bacterial organism along with the gene's preferred name, functional annotation of the gene, and confidence scores. The STRING database offers an Application Programming Interface (API) through which one can retrieve annotations and information about a gene. This API was used to retrieve the preferred names and functions of the gene. The confidence scores stated are the predicted probabilities of the genes to be essential, obtained from the ML model. For a gene to be classified as essential in our model, it should score a predicted essentiality probability of at least 70%. Therefore, the essentiality score runs from 70.0 to 100.0 in the database.

**Figure 2:**
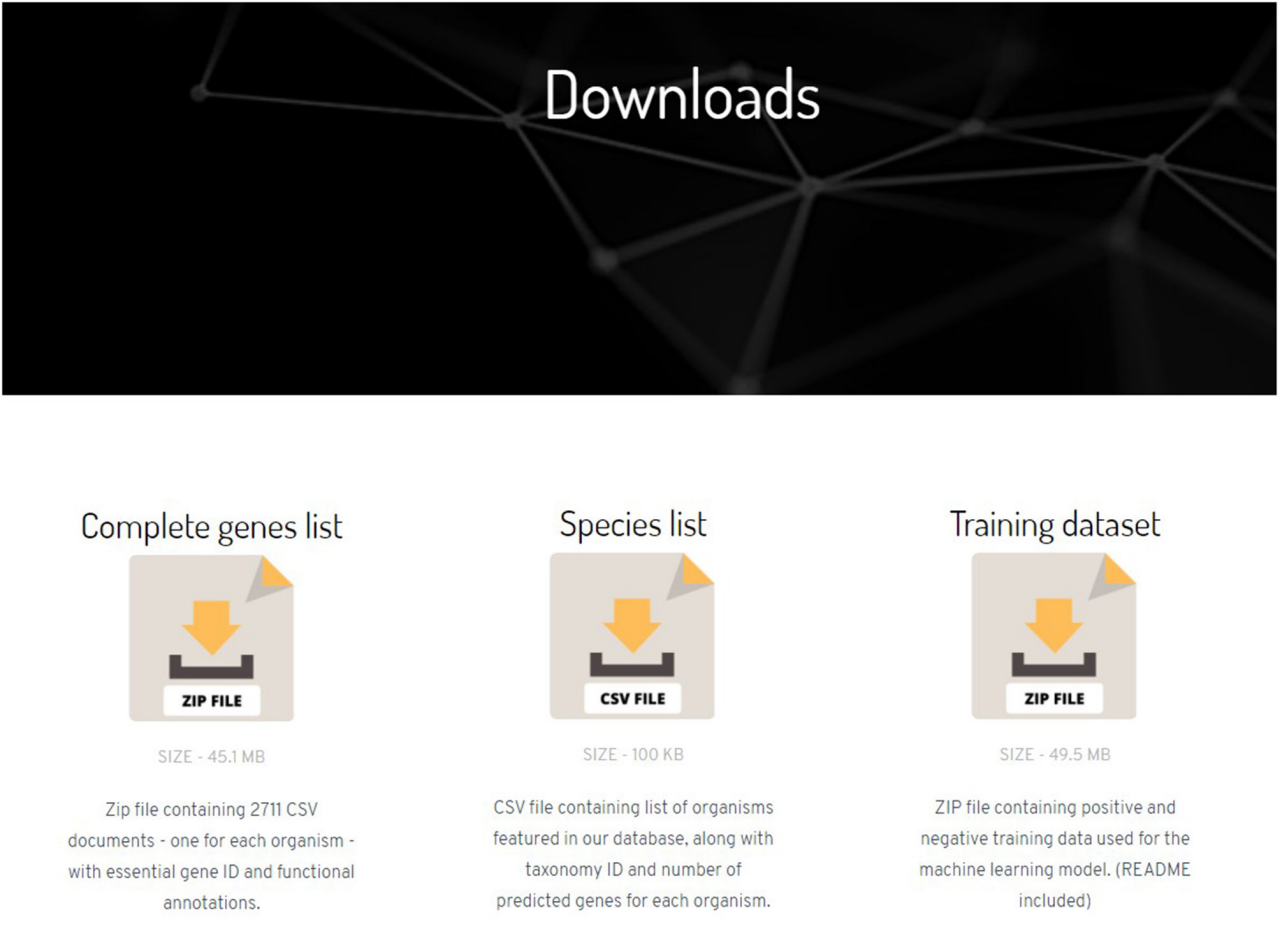
Screenshot of Downloads page in the NetGenes database.

The website also has a “Downloads page” (Figure 2) where the user can download a ZIP file containing all the prediction data along with the annotations and score. Links to download training dataset and feature matrices used in the prediction model can also be found in Downloads page.

## 4. Discussion

Here, we presented a standalone web database called NetGenes, which contains computationally predicted essential genes of 2,711 bacterial organisms. Extending an ML model we previously developed (Azhagesan et al., 2018), we extract network features from as many as 2,711 bacterial interactomes from the STRING database and predict essential genes.

The highlight of this study is that features extracted from protein–protein interaction networks were able to provide good classification capacity between essential and non-essential genes. One important fact to note here is that there is a third class of genes based on essentiality called “fitness genes”, whose essentiality varies depending on the survival conditions. Such genes are not taken into account in our model since there is not enough representation of this third class in order to include it as a separate prediction class. However, as and when sufficient data are available to label genes appropriately, it will be possible to also predict fitness genes by building on the ML models presented here.

Given the extreme paucity of experimentally validated gene essentiality data, the high-confidence predictions generated via this database are likely to be highly useful to experimentalists, for prioritizing genes and generating new hypotheses for experimental validation. The database is easy to access and also provides annotations and ready connections to the STRING database to enable further analyses.

## Data Availability Statement

The datasets presented in this study can be found in online repositories. The names of the repository/repositories and accession number(s) can be found in the article/Supplementary Material.

## Author Contributions

KR conceived the study. VS collected and analyzed data, built the ML models, and developed the web database and drafted the first version of the manuscript. BR and KR supervised the study. All authors revised and approved the final manuscript.

## Funding

VS's work is supported by IBSE. BR's work is partly supported by a Faculty research award from Intel India.

## Conflict of Interest

The authors declare that the research was conducted in the absence of any commercial or financial relationships that could be construed as a potential conflict of interest.

## Publisher's Note

All claims expressed in this article are solely those of the authors and do not necessarily represent those of their affiliated organizations, or those of the publisher, the editors and the reviewers. Any product that may be evaluated in this article, or claim that may be made by its manufacturer, is not guaranteed or endorsed by the publisher.
